# Feasibility of implementing rehabilitation outcomes on a specialist adult critical care unit

**DOI:** 10.1186/2197-425X-3-S1-A161

**Published:** 2015-10-01

**Authors:** S Betteridge, C Bradley, CC Reilly

**Affiliations:** Kings College Hospital, Physiotherapy, London, United Kingdom

## Introduction

There is currently no gold standard rehabilitation outcome measure for Critical Care in the UK. Given the complex nature of these patients, it has been suggested that a 'battery' of outcome measures maybe more appropriate for this setting ([1]). Prior to August 2014 rehabilitation outcome measures were not routinely used on our Adult Liver Intensive Care Unit.

## Objective

To assess the feasibility and usefulness of implementing a battery of rehabilitation outcome measures on an Adult Liver Intensive Care Unit (LITU).

## Methods

A prospective service improvement project. Adult patients with a length of stay greater than 5 days on LITU were included. Three outcome measures were selected for use on the LITU; Rehabilitation Complexity Scale (RCS), ICU Mobility Scale (ICUMS) and The Chelsea Critical Care Physical Assessment tool (CPAx). These outcomes were measured on day 5 of admission, start of rehabilitation and on discharge. Start of rehabilitation versus discharge scores for each outcome measure were evaluated using the paired sample t test and Pearson's correlation analysis.

## Results

Between August 2014 and January 2015, 105 patients were admitted to LITU with a length of stay greater than 5 days and included in this project. The RCS was measured in all patients on day 5 of admission. From the start of rehabilitation to discharge from LITU repeated measures were obtained for RCS = 85 /87 (97%), ICU Mobility Scale = 65/78 (83%) and CPAx = 65/ 78 (83%) of eligible patients.

Reasons for missing data include; RCS (RIP = 13, LITU care ongoing at time of analysis = 5, not recorded = 2), ICUMS and CPAx (RIP= 13, no therapy input required = 9, LITU care ongoing at time of analysis = 5, not recorded = 18).

All measures demonstrated significant improvements with mean SD differences of; RCS = 2.37 (2.78), ICU Mobility Scale = 1.58 (2.13) and CPAx 6.6 (9.26) p= < 0.001. RCS and CPAx at start of rehab were correlated with length of stay (r = 0.27, = 0.29, p < 0.05).

## Conclusions

All measures were feasible to use with the majority of appropriate patients being scored throughout the critical care rehabilitation pathway. All measures demonstrated significant improvements from start of rehabilitation to discharge from LITU showing they are useful measures to use within this setting.
